# Ionic Liquid-Incorporated Zn-Ion Conducting Polymer Electrolyte Membranes

**DOI:** 10.3390/polym12081755

**Published:** 2020-08-06

**Authors:** Jianghe Liu, Sultan Ahmed, Zeba Khanam, Ting Wang, Shenhua Song

**Affiliations:** 1 Shenzhen Key Laboratory of Advanced Materials, School of Materials Science and Engineering, Harbin Institute of Technology, Shenzhen 518055, China; liujianghe@stu.hit.edu.cn (J.L.); usmanisultan@gmail.com (S.A.); zbkhnm@gmail.com (Z.K.); 2 Guangdong Provincial Key Laboratory of Electronic Functional Materials and Devices, Huizhou University, Huizhou 516001, China; twang-hawk@foxmail.com

**Keywords:** polymer electrolyte, Zn-ion conducting, ionic liquid, ionic conductivity

## Abstract

In this study, novel ionic liquid-incorporated Zn-ion conducting polymer electrolyte membranes containing polymer matrix poly (vinylidene fluoride-hexafluoropropylene) (PVdF-HFP) and 1-ethyl-3-methylimidazolium trifluoromethanesulfonate (EMITf), along with zinc trifluoromethanesulfonate Zn(Tf)_2_, are prepared and investigated. It is ascertained that the optimal membrane ILPE-Zn-4 (the mass ratio of EMITf:Zn(Tf)_2_:PVDF-HFP is 0.4:0.4:1), with abundant nanopores, exhibits a high amorphousness. At room temperature, the optimized electrolyte membrane offers a good value of ionic conductivity (~1.44 × 10^−4^ S cm^−1^), with a wide electrochemical stability window (~4.14 V). Moreover, the electrolyte membrane can sustain a high thermal decomposition temperature (~305 °C), and thus its mechanical performance is sufficient for practical applications. Accordingly, the ionic liquid-incorporated Zn-ion conducting polymer electrolyte could be a potential candidate for Zn-based energy storage applications.

## 1. Introduction

Owing to the development of energy storage technology around the globe, mobile electronics have seen a rapid growth. To make our lives more convenient, various electronic products like smartphones, laptops, digital cameras, electric vehicles, etc. have been integrated into our daily life. To date, Li-ion batteries have been widely applied in electronic equipment [1]. However, the uneven geo-distribution of elemental Li and high cost seriously restrict the development of Li-ion batteries [2,3]. Therefore, more abundant and lower-cost metal ions (monovalent cations: Na^+^, K^+^, multivalent cations: Mg^2+^, Zn^2+^, and Al^3+^) are preferred for energy storage systems [4]. Among them, Zn is present in abundance on earth. Furthermore, it has a low cost and a relatively higher volumetric capacity of ~5855 mAh cm^−3^ in comparison to Na (~1129 mAh cm^−3^), Li (~2061 mAh cm^−3^), and Mg (~3834 mAh cm^−3^) [5]. Inspired by these virtues, the Zn-based energy storage system has been drawing considerable attention. Zn-ion batteries usually use Manganese Dioxides, Vanadium Compounds, Prussian Blue Analogs as cathodes and metal Zn plates as anodes [6,7,8]. The aqueous electrolyte solutions of ZnSO_4_, Zn(CH_3_COO)_2_, Zn(CF_3_SO_3_)_2_ have been widely studied in Zn-ion batteries [9,10,11]. However, the work voltage window of aqueous electrolytes is narrow (~1.2 V). Though organic electrolytes offer a high ionic conductivity and wide voltage window, they suffer from other problems like leakage, flammability, corrosiveness, and toxicity [12]. Due to their excellent properties, like a high thermal stability, relatively higher electrochemical stability, high flexibility, and safety, ionic conductive polymer electrolytes have emerged as potential candidates for use in the electrochemical energy storage field [13,14].

In general, on account of the different compositions of the polymer electrolyte system, ionic conductive polymer electrolytes have been divided into two classes. These are solid polymer electrolytes (SPEs) and gel polymer electrolytes (GPEs). For SPEs, the ionic salts are complexed with flexible polymer chains, and the transportation of ions depends on the motion of polymer segments [15]. GPEs are formed by ionic salts and plasticizer mixed into polymer matrices. Due to their high room-temperature ionic conductivity and contact with electrodes with a good compatibility, GPEs have attracted considerable attention [16]. Traditional plasticizers, having a low chemical and electrochemical stability and inflammability, include propylene carbonate (PC), ethylene carbonate (EC), ethyl methyl carbonate (EMC), dimethyl carbonate (DMC), etc. [17,18]. Ionic liquid with excellent electrochemical properties can be a good substitute for traditional plasticizers. For this reason, the imidazolium-based ionic liquid (1-ethyl-3-methylimidazolium trifluoromethanesulfonate, EMITf) was chosen as a plasticizer in the present study. This ionic liquid exhibits a lower viscosity and higher thermal stability [17,19]. Moreover, ionic liquid can also provide a lot of free ions so as to promote the ionic conductivity of polymer electrolytes [20]. In addition to plasticizers, GPEs contain various functional polymer hosts. Generally, these are poly (vinyl alcohol) (PVA), poly(acrylonitrile) (PAN), poly (methyl methacrylate) (PMMA), and poly (vinylidene fluoride-hexafluoropropylene) (PVdF-HFP) [14,21,22,23,24]. Among them, PVdF-HFP with a low crystallinity and high dielectric constant is chosen as the polymer host [25,26,27]. PVdF-HFP consists of both amorphous and crystalline structures. The amorphous region is beneficial to ion transport, while the crystalline structure provides a better mechanical property [28,29,30]. Furthermore, PVdF-HFP exhibits sound chemical and thermal stabilities due to its strong C-F bonds [31,32]. Therefore, in recent years, the ionic liquid-doped PVdF-HFP polymer electrolyte has been studied intensively. Guo et al. [23] developed a polymer electrolyte based on the PVDF-HFP/LiTFSI/SiO_2_/EMITFSI system, and an optimum electrolyte with an ionic conductivity of 0.74 mS cm ^−1^ at 25 °C was applied in a Li/LiFePO_4_ battery. Such a battery showed an excellent cycling stability. Singh et al. [17] fabricated a flexible GPE containing PVdF-HFP and EMIMFSI, along with LiTFSI. The GPE showed the highest ionic conductivity, at ~3.8 × 10^−4^ S cm^−1^ at 25 °C. Kumar et al. [33] reported an electrolyte membrane based on the PVdF-HFP/NaCF_3_SO_3_/EMITf system, and the electrolyte membrane offered an ionic conductivity of ~ 5.7 × 10^−3^ S cm^−1^ at room temperature. Tang et al. [34] revealed that a GPE film of PVdF-HFP:Mg(Tf)_2_ (9:1) doped with 40 wt.% EMITf exhibited a high room-temperature ionic conductivity of ~4.63 × 10^−3^ S cm^−1^ with a wide electrochemical stability window of ~4.8 V. Thus, it was inferred that the ionic liquid-doped PVdF-HFP polymer electrolyte exhibits excellent electrochemical properties.

In the present work, we have prepared ionic liquid-incorporated Zn-ion conducting polymer electrolytes, and studied their structural, electrical, electrochemical, thermal, and mechanical properties. A detailed study on solid polymer electrolytes based on the PVdF-HFP/Zn(Tf)_2_ system was conducted in our previous work [35]. The SPE exhibited a good electrochemical performance when the mass ratio of Zn(Tf)_2_ to PVdF-HFP was 0.4. Hence, we chose the optimal composition to be the host system in the present work. The electrochemical properties of the polymer electrolyte were enhanced by doping ionic liquid. The ionic liquid-incorporated polymer electrolytes were investigated by means of structural, electrical, electrochemical, thermal, and mechanical analyses.

## 2. Materials and Methods

The following materials were used in the preparation of ionic liquid-doped polymer electrolytes and supercapacitors. Poly (vinylidene fluoride-co-hexafluoropropylene) (PVdF-HFP, MW ~40,000), Zinc trifluoromethanesulfonate (Zn(Tf)_2_, purity ~99.6%), and tetrahydrofuran (THF, purity ~99.5%) were acquired from Sigma Aldrich. Ionic liquid 1-ethyl-3-methylimidazolium trifluoromethanesulfonate (EMITf, purity ~99.8%) was obtained from Aladdin. The chemicals were used as received. Both the ionic liquid EMITf and salt Zn(Tf)_2_ were vacuum-dried at 100 °C for 2 h prior to use to remove the moisture.

**Figure 1:**
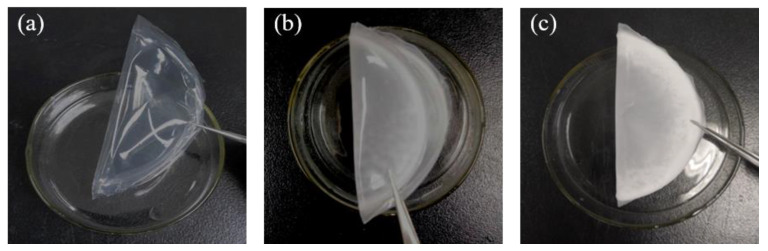
Macroscopic morphologies of the (**a**) SPE-Zn membrane, (**b**) ILPE-Zn-4 membrane, and (**c**) ILPE-Zn-5 membrane.

According to our previous work [30], the solid polymer electrolyte exhibits the best electrochemical properties when the mass ratio of Zn(Tf)_2_ to PVdF-HFP is 0.4, and this composition was chosen as the host system for the preparation of ionic liquid-incorporated Zn-ion polymer electrolyte (ILPE) membranes. Moreover, ILPE membranes with different contents of ionic liquid (the mass ratio of EMITf to PVdF-HFP being 0, 0.1, 0.2, 0.3, 0.4, and 0.5, marked as SPE-Zn, ILPE-Zn-1, ILPE-Zn-2, ILPE-Zn-3, ILPE-Zn-4, and ILPE-Zn-5, respectively) were prepared via a solution cast method. First, the host polymer, PVdF-HFP, was dissolved in THF with the help of magnetic stirring at 50 °C. Subsequently, Zn(Tf)_2_ was added to the solution in a mass ratio of Zn(Tf)_2_ to PVdF-HFP of 0.4, followed by continuous stirring at 50 °C for 2 h. Afterwards, different contents of the ionic liquid EMITf were added, and the mixture was continuously stirred for 18 h to obtain a homogenous viscous solution. Thereafter, the resulting homogeneous solution was poured into different Petri dishes, followed by the evaporation of THF at 35 °C for 10 h. Finally, the obtained free-standing ILPE membranes were stored in a vacuum oven at 40 °C for 4 h, so that the THF could be completely removed. The macroscopic morphology of the obtained electrolyte membrane is shown in Figure 1. It may be noted that the SPE-Zn membrane is uniformly transparent and impurity-free. The membrane becomes translucent with the addition of EMITf (see Figure 1b,c). All the membranes display a good flexibility. The thickness of the membranes was measured to be ~145 μm via a spiral micrometer gauge. The as-prepared electrolyte membranes were stored in a glove box filled with Ar atmosphere.

The crystal structure and crystallinity of the polymer electrolyte membranes were examined using X-ray diffraction (XRD, D/max 2500 PC, Rigaku, Japan) in the 2*θ* range of 5–65° with a 2°/min scan rate. Scanning electron microscopy (SEM, Hitachi S-4700, Tokyo, Japan) was used to observe the morphological characteristics of the electrolyte membranes. The thermal stability of the ILPE membranes was studied by a thermal analyzer (STA 449F3 Jupiter, Selb, Germany) under an Ar atmosphere at a rate of 10°/min between 30 and 600 °C. Using a universal testing machine (CMT7504, Ningbo, China), the mechanical performance of the electrolyte membranes was evaluated. The sample size was: thickness (0.14 mm) × width (10 mm) × gauge length (100 mm). The ionic conductivity analysis for the electrolyte membranes was carried out using a frequency analyzer (PSM 1735, Newton, UK) in the range of 1 Hz to 1 MHz. The electrochemical stability window (ESW) and ionic transference number were evaluated by cyclic voltammetry (CV) and DC polarization method, separately, by means of a CHI760D electrochemical workstation. The scan range was −3 to 3 V with a scan rate of 10 mV s^−1^ for the CV measurements, and the applied voltage was 0.5 V for the determination of the ionic transference number with DC polarization. As for the above electrochemical measurements, the ILPE membranes were assembled in a symmetric sandwich structure cell with stainless steel electrodes (SS/ILPE membrane/SS), in which the electrolyte-electrode contact area was 1.96 cm^2^. Finally, to assess the practicality of an optimal electrolyte membrane as an ionic conductor, it was connected into the circuit.

## 3. Results and Discussion

**Figure 2:**
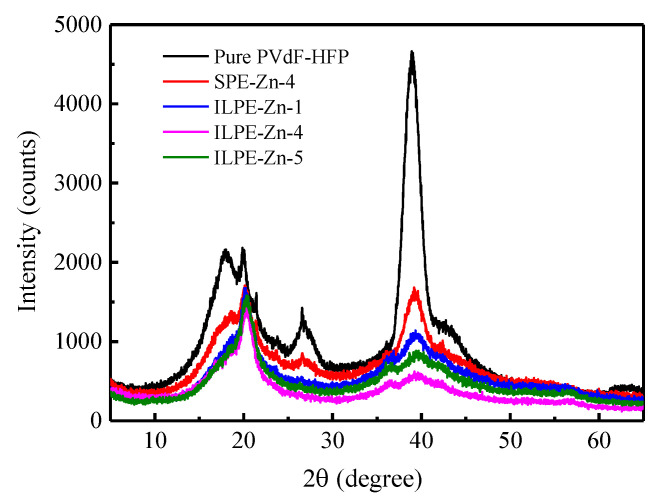
XRD patterns of ILPE membranes with different EMITf contents.

XRD was used to analyze the structure and crystallinity of PVdF-HFP based gel electrolytes. The analyzed results are shown in Figure 2. The pure PVDF-HFP consists of crystalline regions with amorphous domains and presents a typical semi-crystalline characteristic. However, with the addition of Zn(Tf)_2_ to pristine PVdF-HFP, there is a dramatic change in the structure of PVdF-HFP. The intensity of the peaks decreases, and their width increases, thereby expanding the amorphous region of the host polymer [35]. It may be noted that, with the addition of ionic liquid, there is a suppression in the peak intensity. This indicates that the ionic coordination between ionic liquid and polymeric macromolecules with Zn(Tf)_2_ may interrupt the structure of the polymer and weaken the intermolecular interaction of the polymer chains, thereby reducing the degree of crystallinity and thus enhancing the amorphous behavior [34,36]. However, when the addition of ionic liquid is too large, the crystallinity of the electrolyte membranes increases, rather than further decreasing. This may be attributed to the re-association of excess Tf^−^ anions with Zn^2+^ cations, weakening the interaction between ions and polymer segments [37]. It may be noted that, for ILPE-Zn-4, the peak at 2θ ~26.6° is completely suppressed and that, accordingly, the amorphousness of the host polymer has reached its maximum. Moreover, the mobile charge carriers can be transported speedily in amorphous regions in the polymer electrolyte system because they have more free spaces, hereby facilitating the migration of ions [38]. Therefore, it is believed that the ILPE-Zn-4 with larger amorphous regions should be favorable for ion movement.

**Figure 3:**
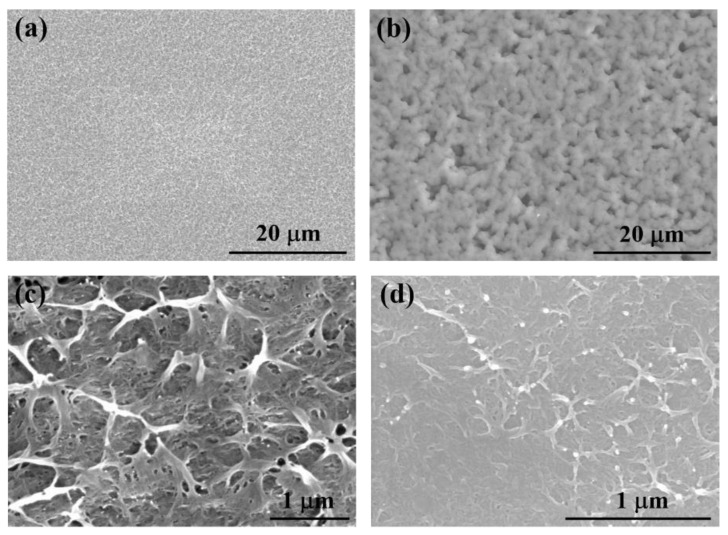
SEM images of (**a**) SPE-Zn, (**b**) ILPE-Zn-4, (**c**) SPE-Zn cross-section, and (**d**) ILPE-Zn-4 cross-section.

Figure 3 displays the SEM images of the ILPE membranes. The surface of the solid polymer electrolyte SPE-Zn can be seen as being wrinkled, with barely visible micropores (see Figure 3a). However, the cross-section morphology of SPE-Zn implies that there are a number of nanopores inside with network structures. For ILPE-Zn-4 (see Figure 3b,d), the addition of ionic liquid EMITf improves the surface morphology in terms of nanopores. It may be noted that the nanopores are interconnected with each other, which may be attributed to the interaction between polymeric macromolecules and ionic liquid, in addition to the evaporation of the THF solvent [39].

There is a significant change in the internal structure of the polymer electrolyte with the addition of ionic liquid. The pores become more abundant, with fine sizes. In general, this is beneficial for ion transportation, as the small interconnected nanopores’ structure and high specific surface areas provide a continuous pathway for ion transportation [34,40,41]. As a consequence, it is speculated that the introduction of ionic liquid to the polymer electrolyte may enhance its ionic conductivity.

**Figure 4:**
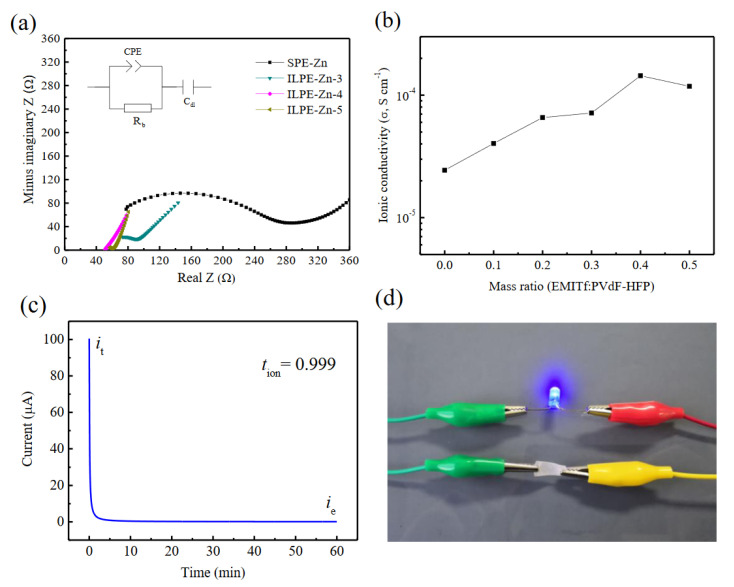
(**a**) Nyquist plots of the ILPE membranes with different EMITf contents measured at ambient temperature and corresponding equivalent circuit (CPE = constant phase element, R_b_ = resistor, and C_dl_ = double-layer capacitor). (**b**) Variation of ambient temperature ionic conductivity with the mass ratio of EMTIf to PVdF-HFP. (**c**) DC polarization curve of ILPE-Zn-4 membrane under 0.5 V at room temperature. (**d**) The ILPE-Zn-4 membrane used as an ionic conductor to successfully connect the LED circuit.

The room-temperature ionic conductivity of polymer electrolytes is one of the vital parameters to consider. AC impedance technique was used to analyze the ion conduction behavior of polymer electrolytes. Figure 4a shows the Nyquist plots of gel electrolyte membranes measured at ambient temperature, and the inset shows their equivalent circuit. It is clear that the plots consist of a quasi-semicircle with a spike line. The quasi-semicircle in the high frequency range is present as a result of the parallel combination of the resistor and constant phase element. The constant phase element may be regarded as a non-ideal capacitor. The spike line located in the low frequency range indicates the formation of a double-layer capacitor at the interface between the electrode and electrolyte [42]. It is noted that the dip angle between the spike line and the real axis is smaller than 90° and arises from the roughness of the electrolyte-electrode interface [43]. The bulk resistance (*R*_b_) of the electrolyte membrane can be determined from the intercept of the semicircle on the real axis. Clearly, the bulk resistance of the polymer electrolyte decreases with the addition of EMITf and exhibits a minimum magnitude when the mass ratio of EMITf to PVdF-HFP is 0.4. Moreover, the high frequency quasi-semicircle almost disappears for a mass ratio of 0.4, indicating that the ILPE-Zn-4 electrolyte prevails in the resistive nature and that its capacitive character is absent [44]. In such a case, the bulk resistance (*R*_b_) is usually obtained from the interception of the inclination spike on the real axis [45,46]. The ionic conductivity of ILPE membranes is given by Formula (1) [47], and the results are listed in Table 1.

(1) $$\sigma =\frac{d}{R_{b}S}$$

where *R*_b_ is the bulk resistance (Ω), *d* represents the membrane thickness (cm), and *S* is the electrolyte-electrode contact area (cm^2^).

Figure 4b depicts the variation of the ambient temperature ionic conductivity with the mass ratio of EMITf to PVdF-HFP. It may be noted that the ionic conductivity of the SPE-Zn membrane without ionic liquids is low, being just ~2.44 × 10^−5^ S cm^−^^1^. However, the ionic conductivity of the ILPE membrane is improved by adding ionic liquid EMITf into the system, and it shows a high value of approximately 1.44 × 10^−4^ S cm^−^^1^ when the mass ratio is 0.4. With the further addition of EMITf, the ionic conductivity decreases. Accordingly, in terms of ionic conductivity, the ILPE-Zn-4 is the best composition. In general, the ionic conductivity (*σ*) depends on three factors: the concentration (*n*_i_), mobility (*q*_i_), and charge (*u*_i_) of mobile ions. The relationship between them can be described by [48]:

(2) $$\sigma =\sum n_{i}q_{i}u_{i}$$

Combined with the above structural analysis, it is believed that the enhancement of the ionic conductivity may be ascribed to both the improvement of the amorphous phase and the increase of mobile ions, caused by the addition of EMITf [17]. However, the addition of excessive ionic liquid would make the ions aggregate, thereby reducing the density of the mobile charge carriers and hindering their movement [39]. Consequently, the ionic conductivity decreases.

In order to confirm that the prepared polymer electrolyte is highly ionically conductive, which is vital for its application, the ionic transference number was determined by DC polarization at room temperature. Figure 4c shows the DC polarization current versus time for the ILPE-Zn-4 polymer electrolyte membrane. Generally, the initial current (*i*_t_) is attributed to the transportation of both ions and electrons. It can be observed that the current decreases dramatically with an increasing polarization time until reaching a steady-state value. This is primarily due to the blockage of ions by the electrodes. Therefore, the final steady-state current (*i*_e_) is merely contributed by electrons. The ionic transference number is calculated by [49]:

(3) $$t_{\mathrm{ion}}=\left(i_{t}-i_{e}\right)/i_{t}$$

The ionic transference number of the ILPE-Zn-4 membrane is obtained as being ~0.999 ± 0.0003. The ultrahigh ionic transference number demonstrates that the conductivity of the polymer electrolyte membrane is mainly caused by ionic species, while the electronic contribution is too small to be considered. Therefore, the present polymer electrolyte membrane is almost 100% ionically conductive. Moreover, the ultrahigh ionic transference number ~0.999 is superior to the ionic transference number reported in literatures, such as the PVdF-HFP/LiBF_4_/EMIMBF_4_ electrolyte (~0.984) [43], PVdF-HFP/LiTFSI/EMIMFSI electrolyte (~0.99) [50], and PVA/LiTFSI/EMITFSI electrolyte (~0.995) [37]. To demonstrate the practicability of the polymer electrolyte, a closed-loop circuit composed of a LED bulb with the ILPE-Zn-4 electrolyte membrane was designed. The LED bulb was successfully lit and emitted a brilliant blue light (see Figure 4d). This phenomenon further indicates that the obtained ILPE-Zn-4 polymer electrolyte is an ionic conductor.

**Figure 5:**
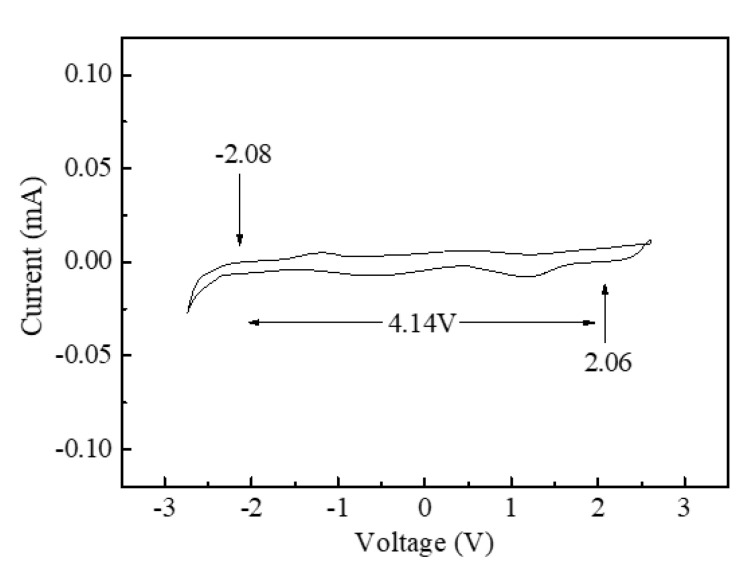
CV curve of the ILPE-Zn-4 membranes at a scan rate of 10 mV s^−1^.

The electrochemical stability of the ILPE membrane is essential for its practical applications. Figure 5 displays the CV curves of the ILPE-Zn-4 electrolyte membranes at a scan rate of 10 mV s^−1^. One can see that the current is stable within the potential range of −2.08 to 2.06 V (ESW: ~4.14 V), which signifies that the electrolyte membrane can work stably within this potential range. The current rising beyond this range may relate to the degeneration of the electrolyte membrane, manifested by the occurrence of an electrochemical reaction. Nevertheless, this ESW (~4.14 V) is acceptable for electrochemical device applications. In addition, there are a pair of small current humps at around ±1.3 V, which probably arise from the formation of ion pairs or other by-products during this process [51,52].

**Figure 6:**
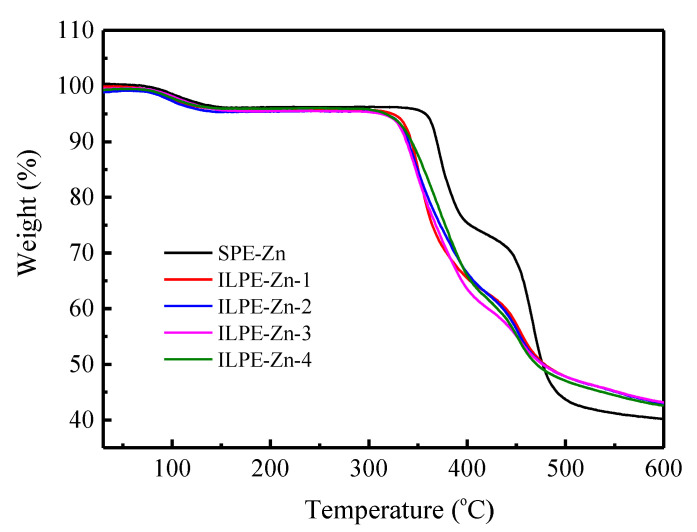
TGA curves of the ILPE membranes with different EMITf contents.

Thermal stability is a significant parameter for a polymer electrolyte membrane. A low thermal decomposing temperature in the polymer electrolyte may give rise to electrolyte membrane degradation in a short circuit, which may produce substantial heat accumulation. Hence, the electrolyte membranes should possess an excellent thermal stability. Figure 6 illustrates the TGA curves for ILPE membranes. It may be clearly observed that all polymer electrolytes show a slight drop between 80 to 180 °C. The weight losses of the membranes are: SPE-Zn ~4.07%, ILPE-Zn-1 ~3.93%, ILPE-Zn-2 ~3.80%, ILPE-Zn-3 ~4.03%, and ILPE-Zn-4 ~3.55%, which may be attributed to the evaporation of water absorbed in the ILPE membranes. However, a considerable decomposition of the EMITf-free SPE-Zn membrane may be observed when the temperature exceeds 350 °C. With the addition of ionic liquid, the polymer electrolyte membrane’s decomposition temperature is lowered. This is primarily because of the enhancement of the polymer chain flexibility caused by the interaction of ionic liquid and Zn salt with the host polymer [53].

Despite this, the ILPE membranes incorporated with ionic liquid still display a sufficiently high thermal stability, and the decomposition temperature is approximately 305 °C. Therefore, in terms of the thermal stability, the polymer electrolyte membranes can be considered suitable for energy device applications.

**Figure 7:**
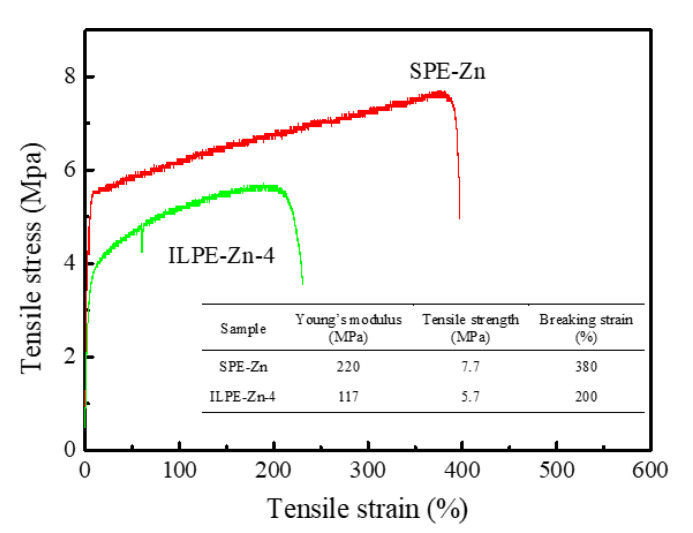
Typical stress-strain curves of SPE-Zn and ILPE-Zn-4 membranes and their mechanical properties (Young’s modulus, tensile strength, and breaking strain).

In addition to a good thermal stability, the mechanical performance is also an imperative characteristic of electrolyte membranes. The typical stress-strain curves of SPE-Zn and PE-Zn-4 electrolyte membranes are shown in Figure 7. The values of the Young’s modulus, tensile strength, and breaking strain are listed in the inset table. The Young’s modulus and tensile strength of SPE-Zn are 220 MPa and 7.7 MPa, respectively, while the breaking strain is 380%. By contrast, those of ILPE-Zn-4 fall down to 117 MPa, 5.7 MPa, and 200%, respectively.

The mechanical performance declines with the addition of EMITf. This may be due to a more amorphous phase and porous structure, created by the addition of EMITf in the ILPE-Zn-4 electrolyte membranes. In general, the polymer chains are highly flexible in the amorphous domains, thus reducing the interaction force between polymer molecules. Nevertheless, the electrolyte membranes still exhibit sufficient mechanical properties. This phenomenon has also been confirmed by other studies. As reported by Tang et al. [34], the electrolyte membrane (90PVdF-HFP:10Mg(Tf)_2_ + 40EMITf) fabricated by solution casting possesses the following mechanical properties: Young’s modulus = ~66 MPa, tensile strength = ~3.4 MPa, and breaking strain = ~633%. A study by Jie et al. [54] indicates that the tensile strength and breaking strain are about 2.2 MPa and 250%, respectively, for the PVDF-HFP/LiTFSI/NMP gel electrolyte film. Obviously, our values are highly comparable to theirs. In addition, Can et al. [55] developed a solid polymer electrolyte based on TPU/PEO = 1:3, showing that the electrolyte had a superior comprehensive performance with a tensile strength of 1.38 MPa and that it was successfully applied to the LiFePO_4_/SPE/Li battery in the temperature range of 60 to 80 °C. Therefore, the mechanical performance of the ILPE-Zn-4 membrane should be good enough to be eligible for applications in energy storage devices.

## 4. Conclusions

Flexible ionic liquid-incorporated Zn-ion conducting polymer electrolyte membranes were prepared and characterized. Investigations indicate that the addition of ionic liquid EMITf reduces the crystallinity, enriches the nanopores’ structure, and enhances the electrical and electrochemical properties of the electrolyte membranes. With a high thermal stability (thermal decomposition temperature ~305 °C) and good mechanical performance (tensile strength ~5.7 MPa), the optimized polymer electrolyte ILPE-Zn-4 (EMITf: Zn(Tf)_2_: PVdF-HFP = 0.4: 0.4: 1 in mass) exhibits a high ionic conductivity (~1.44 × 10^−4^ S cm^−1^) at an ambient temperature, with a wide electrochemical stability window (~4.14 V). Moreover, the ionic conductive polymer electrolyte exhibits an ultrahigh ion transfer number ~0.999. Therefore, the polymer electrolyte can be used as an ionic conductor to connect the circuit. The properties of the ionic conductor polymer electrolytes demonstrate that the optimized ionic liquid-incorporated Zn-ion conducting polymer electrolyte shows a promising perspective for energy storage applications.

## Figures and Tables

**Table 1:** Ionic conductivities of polymer electrolyte membranes at room temperature.

Samples	Ionic Conductivities (S cm^−1^)
SPE-Zn	2.44 × 10^−5^
ILPE-Zn-1	4.03 × 10^−5^
ILPE-Zn-2	6.56 × 10^−5^
ILPE-Zn-3	7.15 × 10^−5^
ILPE-Zn-4	1.44 × 10^−4^
ILPE-Zn-5	1.18 × 10^−4^
